# The development of episodic foresight in preschoolers: the role of socioeconomic status, parental future orientation, and family context

**DOI:** 10.1186/s41155-019-0125-4

**Published:** 2019-06-15

**Authors:** Alejandro Vásquez-Echeverría, Clementina Tomás, Orlanda Cruz

**Affiliations:** 1 Instituto de Fundamentos y Métodos en Psicología, Facultad de Psicología, Universidad de la República (UdelaR), Tristán Narvaja 1674, 11300 Montevideo, Uruguay; 2 Faculdade de Psicologia e de Ciências da Educação, Universidade do Porto, Porto, Portugal

**Keywords:** Episodic foresight, Consideration of future consequences, Family environment, Cognitive development, Child poverty

## Abstract

Episodic foresight (EF) refers to the ability to anticipate future states of the self. Despite almost two decades of research, no studies explored how family context variables relate to the development of this ability. The objectives of this study were to explore the association of socioeconomic status (SES), parental consideration of future consequences (CFC), and family environment quality on the development of episodic foresight and to compare the magnitude of the effects of these same variables on delay of gratification and planning.

Sixty-four dyads composed by 4-year-old Uruguayan children and their main caregiver participated in the study. Children were administered experiments on episodic foresight, delay of gratification, planning, and receptive language. Parents reported socioeconomic status, family environment, and their consideration of future consequences. Even though parents’ limit setting was associated to higher EF in children and parental CFC-I was a predictor in multiple regression analysis, these effects ceased to be significant when controlled by child’s receptive language and caregiver education, being these the main predictors of EF. Results also indicate that SES significantly distinguishes the performance in future-oriented skills and language, being the magnitude of the effect higher for EF in comparison with planning and delay of gratification. This study supports that EF is related to SES to a greater extent than other variables traditionally assessed in studies of poverty and child development. We discuss implications of low SES and language skills in the light of EF development and immediate-oriented behavior in contexts of deprivation.

## Background

Desire for immediate gratification and present orientation are more frequently observed in adults coming from low socioeconomic backgrounds (Adams & White, 2009; Haushofer & Fehr, 2014; Pepper & Nettle, 2017). However, few studies have related socioeconomic and other family context variables with the development of episodic foresight (EF) in young children. Mapping the variables that contribute to the development of EF may be crucial to understand the intergenerational reproduction of the behavioral constellation of deprivation, as suggested by Pepper and Nettle (2017).

EF is defined as the ability to project oneself into the future to anticipate and pre-experience events, desires, or mental states (Atance & O’Neill, 2001). EF demands the construction of mental representations upon facts that may happen and implies the anticipation of the self in relation to personal future, detached from the actual emotional state or beliefs of the person, and located at an approximate time and place (Atance & O’Neill, 2001; Suddendorf & Redshaw, 2013).

Assessment of EF in young children presents methodological challenges since adult methods are not suitable (e.g., self-report). For this reason, most common methods for evaluating young children’s EF skills include a variety of experimental tasks (verbal, choice, and location tasks; for a review see Hudson, Mayhew, & Prabhakar, 2011), while the main debate is how thoroughly EF is actually being measured as well as the extent to which the construct is isolated from other cognitive demands of the task (e.g., language, working memory, planning).

This concern is also related to how EF is associated with other variables of cognitive development. Hypothesis in specialized literature include the notion of EF as a self-projection process (Buckner & Carroll, 2007) and EF as part of an episodic cognition system, including the role of language in episodic memory and foresight development (Suddendorf, Addis, & Corballis, 2009). EF is also considered in line with other future-oriented processes since it shares a forecasting component with these forms of future-oriented thought such as formal planning and delay of gratification. However, EF is also distinguishable from these. For instance, certain forms of planning that imply making predictions about the physical world (e.g., mentally represent alternative actions or transformations over objects) do not involve envisioning the future self at a specific moment (Jackson & Atance, 2008). For the case of delay of gratification, EF may contribute to performance (e.g., being able to envision the delayed reward may help to discount less the future), even though it has been argued that semantic knowledge and executive functions prevail (Hanson, Atance, & Paluck, 2014; Hudson et al., 2011; Jackson & Atance, 2008; Vásquez-Echeverría, 2015).

EF emerges between 3 and 5 years of age (Atance & Jackson, 2009; Suddendorf & Redshaw, 2013), being, in general, incipient at age 3, presenting considerable individual variability at the age of 4, and improved performance at age 5. This pattern is observed even when different task formats are involved (Bélanger, Atance, Varghese, Nguyen, & Vendetti, 2014; Busby & Suddendorf, 2005; Suddendorf & Busby, 2005). However, to our knowledge, no studies have explored hypotheses regarding the interindividual differences in EF found at this developmental period.

Interindividual differences in cognitive development are explained, to an important extent, by characteristics of developmental contexts and the proximal processes to which children are exposed (Bronfenbrenner & Morris, 2006). Given the absence of studies that systematically analyze interindividual differences in EF, we will examine the association of family context variables (family SES and environment quality, and caregiver future orientation) that given their influence on general cognitive development, we expect will also affect EF and the cognitive demands associated to foresight. One of the variables that is most related to cognitive and socioemotional development is SES of the family. Low SES has been associated to school dropout, lower scores on IQ measures, and executive functioning tasks, among others (Bradley & Corwyn, 2002; Gottfried, Gottfried, Bathurst, Guerin, & Parramore, 2012). Causes underlying this phenomenon include adverse effects of persistent poverty on parental investment, higher stress levels, and the absence of material resources for stimulation (Dickerson & Popli, 2016; Evans & Kim, 2013).

Other contextual factors that have been reported to explain the influence of SES in child development include quantity and quality of stimulation that a child receives at home, e.g., interaction and linguistic stimulation, even before age three (Hart & Risley, 2003); demands of maturity; parental stress and exposure to conflict; care and monitoring; or limit setting, among others (Bradley & Corwyn, 2002; Evans & Kim, 2013). Lastly, some scholars have shown evidence of intergenerational transmission of future orientation in parent-adolescent dyads (Andre, van Vianen, & Peetsma, 2017; Seginer, 2005), but to our knowledge, no studies have explored this phenomenon in parent-child dyads. We believe parental future orientation, such as their level of consideration of future consequences (CFC), could be related to individual differences in future cognition in young children. Hudson (2006) has suggested that the kind of parental linguistic stimulation and the involvement in patterns of future-oriented interaction favor future understanding of the child. Moreover, parents differ in how they share future and past experiences to their offspring, and that is related to adolescents’ time perspective (Shirai & Higata, 2016). In this line, it is possible to reason that parents’ CFC level may be determining the temporal horizon of their actions and the type of interaction and temporal language addressed to their children, which in turn, behaviorally models the acquisition and development of EF in their children. Also, lower CFC scores are present, on average, in persons coming from lower SES backgrounds (Adams & White, 2009; Pepper & Nettle, 2017). Nevertheless, to the best of our knowledge, no studies have empirically explored the relations of parental CFC with their offspring levels of EF, in a sample coming from different socioeconomic backgrounds.

### This study

This study has two main objectives. The first is to explore the association of SES, parental CFC, and quality of family environment on EF in a sample of preschool-aged children coming from low and medium-high SES backgrounds. We expect that children from higher SES backgrounds, with better quality of family context and parents with higher CFC scores, will present higher scores on EF tasks. Given that in previous literature, SES independently predicted the variance in cognitive development, quality of family environment, and future orientation, parental education as a continuous measure of objective SES (American Psychological Association, 2007) will be controlled for.

Second, we want to compare the magnitude of the effects found in our first objective with those of CFC, family environment, and SES on delay of gratification and planning. As we mentioned, EF may be related to future-oriented processes, as they all share the demand of acting now to reach a future outcome. Planning and delay of gratification are considered future-oriented processes (Atance & Jackson, 2009) that present a weaker development in children from low SES backgrounds (e.g., Hackman, Gallop, Evans, & Farah, 2015; Raver, Blair, & Willoughby, 2013). In this sense, it is important to measure if the relation of SES with EF development resembles to the more documented relation between SES and the other future-oriented processes. Since these processes are theoretically related, we expect that the effects of SES, parental CFC, and family context on EF development will be similar when compared to planning and delay of gratification.

## Method

### Participants and procedure

Sixty-four 4-year-old children (31 girls), living in Montevideo, Uruguay, participated in this study, 30 belonging to families of low SES (*M*_age_ = 54.8 months, SD = 3.6) and 34 belonging to families of medium-high SES (*M*_age_ = 53.2 months, SD = 3.7). After obtaining the approval of the National Administration of Public Education of Uruguay (ANEP), four public centers classified as *quintile 1* and four private centers were contacted by convenience. Quintile classification of schools in Uruguay is based on socioeconomic indicators of the families of students attending that school. Families of children from quintile 1 centers had lower scores in all socioeconomic indicators, such as mother education, father education, rooms at home, income, and persons per room, as compared to their private center counterparts who had a medium-high SES family background (see Table 1 for frequencies on main indicators). The main caregivers of children, defined in the first part of the survey as the parent or relative that spends more time in charge of the child, received an invitation to participate in the study along with informed consent (acceptance rate = 61%). The assessment of 10 additional children, from both SES groups, was not completed due to fuzziness, shyness, or experimental error (e.g., incorrect wording). The session, held during school hours, lasted about 30 min. Children completed the experimental tasks (EF, delay of gratification, planning measures) and the PPVT-III. The main caregiver (self-assigned within each family) completed the self-reported questionnaires that were sent home: the sociodemographic and family background questionnaire, the Extadi-Gangoiti scale, and the consideration of future consequences scale. Uncompleted questionnaires were recovered through telephone contact.

**Table 1:** Characteristics of the families participating in this study

	Private centers	Quintile 1 public centers
Parents age, years		
Mother (*M*, SD)	36.2 (5.1)	29.9 (5.5)
Father (*M*, SD)	37.9 (5.9)	32.9 (6.2)
Monthly income, USD		
Less than 750 (*n*)	0	22
750–1000 (*n*)	0	7
1000–1500 (*n*)	17	1
1500–2000 (*n*)	10	0
More than 2000 (*n*)	7	0
Children in the family		
*M* (SD)	2 (0.9)	2.4 (1.2)
One (*n*)	10	6
Two (*n*)	17	11
Three or more (*n*)	7	13
Higher educational level		
Mother		
Elementary (*n*)	0	14
High school (*n*)	10	15
University/other tertiary(*n*)	24	1
Father		
Elementary (*n*)	0	13
High school (*n*)	17	13
University/other tertiary (*n*)	16	1
Main caregiver		
Mother (*n*)	33	28
Father (*n*)	1	1
Grandmother (*n*)	0	1

*SD* standard deviation. Data from three fathers was not reported

### Measures

#### Episodic foresight

##### Draw task

This task evaluates the implication of self in planning (Atance & O’Neill, 2005). First, the experimenter explained that the game consisted in drawing freely, following two rules that demanded self-anticipation: (a) the child had to use the sheets that contained previously designed patterns to be included in the drawing and (b) the child had to previously state what he was going to draw. Scores were computed as follows: 0 (no drawings were achieved), 1 (one drawing was achieved), and 2 (both drawings were achieved according to the initial statement). Coders reached an interrater agreement of *k* = .94, based on a common analysis of 79% of the cases, considered very good according to El Emam (1999). Disagreements were resolved by discussion.

##### Trip task

Atance and Meltzoff’s (2005) trip task was adapted. The child was asked to help his parents prepare a trip. A sequence of photos of places where people would travel to was presented. After the identification of the place represented by the picture, a series of three objects were presented, one of which was relevant to address a future need during the trip. The other two were distractors, one of them semantically related to the place represented by the photograph. The child had to choose an object to take to the trip and explain the choice. One point per correct target item was assigned, plus another point for verbal responses that included the following: (a) a state of the self and (b) future value in its semantic formulation.

##### Composite measure of EF

The composite measure of EF was obtained by adding the score in the trip task and in the draw task. The correlation between scores was rho = 0.38, suggesting its adequacy to collapse the data.

#### Delay reward

##### Delay reward task

Mischel, Shoda, and Peake’s (1988) delay reward task was adapted. The child was offered a sweet (of choice). Then, he was told that he could eat the candy at that moment, but if he waited for the experimenter to return, he would get three more. The child was given a wireless electronic bell to call the experimenter when he wanted to eat the candy. The maximum waiting time was 8 min. Waiting minutes were computed as the score.

#### Planning

##### Tower of Hanoi

Carlson, Moses, and Claxton’s (2004) task was adapted. It presents a wooden structure with three stakes and three disks. For better understanding, the task was presented in a playful way: the stakes represented trees, the biggest disk was “daddy monkey,” the medium disk was “child monkey,” and the smallest disk was “baby monkey.” Before starting, the three rules of the game were explained: (a) the disks (monkeys) must remain in the stakes (trees), (b) only one monkey can jump at a time, and (c) a bigger monkey cannot sit down on a smaller monkey (i.e., no bigger disk can be placed on a smaller disk on the same stake). Then, the experimenter would say to the child: “Now you have to put the monkeys in a position like the one I have” while manipulating his own set of the game to show the position to be reached. The score range was 0 to 6.

#### Receptive vocabulary

##### Peabody

The Peabody Picture Vocabulary Test (Dunn, Dunn & Arribas 2006) is a standardized receptive vocabulary test, used to assess verbal aptitude. This measure is frequently used as a global indicator of cognitive development in children (Beres, Kaufman, & Perlman, 2000). In this test, subjects respond to target words presented verbally, by pointing to one of the four images shown. We used the age-based standard score (*M* = 100, SD = 15) based on the sample of Dunn et al. (2006). Since there is no validated version for the Uruguayan population to date, the available Spanish adaptation was used.

#### Socioeconomic background

##### Socioeconomic status and family background questionnaire

Parents answered a questionnaire on sociodemographic aspects and child data (e.g., occupational and educational level of the parents, household income). The socioeconomic status was operationalized as follows: (1) for the comparison of groups, it was coded according to the context of the child’s educational center (0 = low, 1 = medium-high); (2) for correlations and regression analyses, the educational level was assumed as a continuous variable of ESE (APA, 2007), encoding the academic level reached by the main caregiver (0 = no school studies, 2 = primary studies completed, 4 = basic secondary studies competed, 6 = high school/technical school studies completed, 8 = complete university).

#### Quality of family context

##### Etxadi-Gangoiti scale

The Etxadi scale evaluates different aspects of the quality of the family context (Arranz, Olabarrieta, Manzano, Martín, & Galende, 2014). Self-reported items of three subscales of the Etxadi scale were used: (a) limit setting and strengthening of resilience (alpha = .72), (b) exposure to parental conflict (alpha = .70), and (c) parental stress (alpha = .60). It was completed by the parents, using a Likert scale (1 = never, 6 = very frequently). Higher scores reflect lower quality of the family context for each subscale. This scale presents satisfactory psychometric properties (Arranz et al., 2014; Baigorri, 2015).

#### Consideration of future consequences

##### Consideration of future consequences scale

Parents answered the consideration of future consequences scale (Vásquez-Echeverría, Antino, Alvarez-Nuñez, & Rodríguez-Muñoz, 2018), which assesses the extent to which people consider the future consequences of their actions to determine their behavior. It has two subscales: CFC-Immediate (CFC-I) and CFC-Future (CFC-F), composed of seven items each, and answered in a 7-point Likert-type scale. Higher scores reflect greater consideration for the immediate or distant consequences of actions, respectively. Vásquez-Echeverría et al. (2018) reported good reliability (omegas of .81 for CFC-I and .72 for CFC-F) and good fit of the confirmatory two-factor solution (*χ*2 = 113.97, *df* = 75, CFI = .92, TLI = .90, RMSEA = .05, SRMR = .06).

### Database treatment and analysis plan

Outliers were not found (*Z* < 3). Concerning distribution, most of the variables were normally distributed. Only Extadi-Gangoiti subscales of limit setting and family conflict were skewed higher than 1, and all Extadi-Gangoiti and delay reward scores presented kurtosis higher than 1.5 (Shapiro-Wilk, *p* < 0.01). Adopting a conservative approach, we homogenized the analysis plan using non-parametric statistics for group comparison and correlational analyses. Therefore, Spearman’s correlations and Mann-Whitney *U* tests were performed. Effect size was analyzed through rank-biserial correlations.

Further analyses were performed through regressions. For multiple linear regressions, we visually inspected the p-p plot for normality in the distribution of residuals and the scatterplot of the residuals to verify homoscedasticity. We found that they were satisfactory. Furthermore, data did not present multicollinearity (VIF < 5). Analyses were performed using the softwares JASP 0.9 and SPSS v22.0.

Alpha value was set in 0.05 to interpret statistically significant results for omnibus tests. Following Ferguson’s (2009) guidelines, effect sizes of correlational type are interpreted in this way: .20 as a low effect, .50 as a moderate effect, and .80 as a strong effect.

## Results

We present the results in two sections. First, we analyze the descriptive statistics and correlations among the study variables. In particular, we explore the association of child SES background on study variables via two analyses: a group comparison and a logistic regression. Second, we explore the effects of family context variables on children scores in future-oriented measures through a hierarchical regression. In these analyses, we both analyze the effect of family context variables (including SES) on EF (objective 1) and compare these effects to those found for DR and ToH (objective 2).

### Descriptive statistics and associations

Table 2 presents descriptive and correlational statistics for the entire sample. EF shows moderate and moderate-high correlations with the other child variables (ToH, DR, and PPVT). Negative and significant correlations between child’s variables (specifically EF, ToH, and PPVT) and the parental CFC-I scores are observed. There is also a negative correlation pattern between difficulty in limit setting and developmental variables (statistically significant for ToH and DR). We controlled for sex differences and only found statistically significant differences in delay of gratification, in favor of girls (*W* = 344.5, *p* = .020, rank-biserial = 0.33).

**Table 2:** Descriptive statistics, group comparison results by SES, and Spearman’s correlations between measures

	Descriptives			Comparison		Correlations							
	Tot	SES-MH	SES-L	U	ES	2	3	4	5	6	7	8	9
1. EF.	4.27 (2.4)	5.65 (1.74)	2.7 (2.1)	147.5***	0.71	.31*	.37**	.59**	− .28**	.06	− .15	.30*	− .23
2. ToH	2.84 (1.69)	3.52 (1.4)	2.07 (1.7)	295.0**	0.42	–	.48**	.42**	− .30*	.05	− .27*	.17	− .25*
3. DR	4.06 (3.8)	5.6 (3.1)	2.37 (3.4)	243.5***	0.52		–	.42**	− .23	.09	− .26*	.28*	.03
4. PPVT	102.4 (17.8)	112.5 (12)	90.5 (16.3)	139.0***	0.72			–	− .30*	.10	− .19	− .03	− .34*
5. CFC-I	2.87 (1.27)	2.40 (1.0)	3.40 (1.43)	291.0**	− 0.43				–	− .11	.09	.08	.46**
6. CFC-F	4.56 (1.42)	4.68 (1.20)	4.4 (1.63)	472.5	0.07					–	− .24	.04	.29*
7. EG-L	1.90 (0.73)	1.77 (0.59)	2.05 (0.85)	400.5	− 0.22						–	.08	.05
8. EG-C	1.60 (0.66)	1.65 (0.67)	1.55 (0.66)	450.5	0.12							–	.24
9. EG-S	2.70 (1.01)	2.33 (0.58)	3.11 (1.22)	295.0**	− 0.42								–

Standard deviation between parenthesis*. SES-MH* medium-high SES dyads, *SES-L* low SES dyads, *1* episodic foresight, *2* Tower of Hanoi, *3* delay reward, *4* Peabody Picture Verbal Test, *5* consideration of future consequences-immediate, *6* consideration of future consequences-future, *7* Etxadi-Gangoiti scale-limit setting; *8* Etxadi-Gangoiti scale-exposure to family conflict, *9* Etxadi-Gangoiti scale-parental stress, *U* Mann-Whitney *U* test, *ES* effect size (rank-biserial correlation)

*p < .05

**p < .01

***p < .001

There were statistically significant differences among all child development variables according to SES background (see Table 2). The effect size of SES background on EF scores was moderate-to-high. Regarding family context variables, CFC-I and parental stress presented statistically significant differences in favor of medium/high SES families (they were less immediate oriented and less stressed).

Given the moderate-to-high effect sizes by SES background in all cognitive development variables and the moderate-to-high correlations between them, we performed a logistic stepwise regression analysis (low SES = 0, medium-high SES = 1) with cognitive variables as predictors. This analysis allowed us to determine which of these variables better characterize children’s membership to each SES group, controlling for their shared contribution. Used in this way, the logistic regression parallels a discriminant analysis but for dichotomous known categories. The resulting selected variables are the most representative of (i.e., are more associated to) the known category*.* PPVT and EF composite were the most relevant variables to characterize child cognitive variables in low versus medium-high SES backgrounds. Results for this two-variable selected model were as follows: Nagelkerke pseudo *R*^2^ = .58, *χ*^2^ (df) = 36.60 (2), *p* < .001 with PPVT, *B* = .07, odds ratio = 1.08, EF composite score with *B* = .49, and odds ratio = 1.65. This means that for a unit of increase in EF composite, the odds of belonging to the medium-high SES group increase by a factor of 1.65, holding the other variable constant. The percentage of correct classification with this model was 86%.

### Hierarchical linear regressions of contextual variables on future-oriented variables

To facilitate the specification of the model, we first performed a regression (stepwise) of the family environment variables (difficulty in setting limits, parental stress, and family conflict) and caregiver’s CFC (immediate and future) on future-oriented variables. CFC-I was a significant predictor of EF composite (*b* (SE) = − 0,51 (0,23), *β* = − 0,27) and planning (*b* (SE) = − 0,39 (0,15), *β* = − 0,29). The score in difficulty in setting limits was a significant predictor in DR models (*b* (SE) = − 1,41 (0,60), *β* = − 0,29) and planning (*b* (SE) = − 0,65 (0,27), *β* = − 0,28). All models were statistically significant in the change of *R*^2^. Based on this criterion, we selected these variables in the specification of the final model. Subsequently, we performed hierarchical multiple linear regression analysis of the contextual variables on EF tasks and related competences (see Table 3). In step 1, we controlled for PPVT and gender. In step 2, we introduced contextual variables, e.g., mother education, CFC-I, and difficulty in limit setting. Results indicate that after being controlled for, there were no contextual predictors that contributed significantly to the model, except for mother education in EF model.

**Table 3:** Hierarchical linear regression with direct scores of PPVT and sex (step 1) and CFC-I and limit setting difficulties (step 2) as predictors of EF, delay reward, and Tower of Hanoi scores

	EF			DR			ToH		
	*b* (SE)	*β* entry	*β* final	*b* (SE)	*β* entry	*β* final	*b* (SE)	*β* entry	*β* final
Step 1									
PPVT	.08 (.01)	.60***	.36**	.08 (.02)	.41***	.24	.04 (.01)	.44***	.20
Sex (girl = 1)	.24 (.49)	.05	.02	2.74 (.81)	.39**	.37**	.69 (.38)	.21	.14
Step 2									
Moher Ed	.44 (.17)	0.35*		.46 (.27)	.25		.23 (.13)	.26	
CFC-I	− .07 (.21)	− 0.04		− .14 (.33)	.05		− .14 (.16)	− .11	
EG-L	− .07 (.35)	− 0.02		− .55 (.55)	− .11		− .35 (.27)	− .15	
Model	*R*^2^ = .44			*R*^2^ = .36		*R*^2^ = .32			

*EF* episodic foresight, *DR* delay reward, *ToH* Tower of Hanoi, *PPVT* Peabody Picture Verbal test-direct score, *Mother Ed* maternal education, *CFC-I* consideration of future consequences-immediate, *EG-L* Etxadi-Gangoiti scale-limit setting subscale

*p < .05

**p < .01

***p < .001

## Discussion

The objectives of this research were (a) to explore the association of SES, parental CFC, and quality of family environment on child’s EF and (b) to compare the strength of the effects of CFC, family environment, and SES on the development of EF with the strength of the effects of CFC, family environment, and SES on delay of gratification and planning, in a sample of Uruguayan preschool-aged children coming from low and medium-high SES backgrounds.

Regarding the first objective, although correlations suggest that some family context variables are associated with higher EF in children and that parental CFC-I was a predictor in the hierarchical multiple regression analysis, these effects ceased to be significant when controlled by linguistic development and maternal education. Despite the theoretical arguments that led us to expect family context variables and parent’s future orientation to be associated to EF development (e.g., self-regulation and inhibitory implications of limit setting, behavioral models based on the temporal horizon of parents’ actions), the exclusion of effects may be due to the fact that their contribution was overlapped to mother’s educational level. In fact, parental CFC-I scores showed significant differences between groups according to SES. Still, the main predictors of EF are child’s receptive language and mother’s educational level. This can be explained considering that (a) language plays a key role in the development of declarative memories, allowing the manipulation of semantic and episodic memory information, thus sustaining the simulation of personal future projections (Suddendorf & Redshaw, 2013), and (b) SES determines differentiated trajectories in the linguistic development of children (Hart & Risley, 2003). This association is also in line with other studies that relate language and temporal cognition development, in particular mother’s elaborative/advanced language related to children’s contributions to future talk and their understanding of the future (Hudson, 2006).

It should also be noted that this study replicates results that relate low SES to higher immediate orientation (Adams & White, 2009; Pepper & Nettle, 2017), observable in higher parental CFC-I and lower EF of the children. The tendency towards immediate orientation in families of low SES could be rooted on the perceptions of scarce control over future outcomes, greater salience of present risks, and enhanced temporal discounting (Haushofer & Fehr, 2014; Pepper & Nettle, 2017). This model could be tested in future studies using structural models of mediation: low SES would produce both a reduction in linguistic stimulation of the child (which implies impoverished use of temporal language), as well as a model of immediate-oriented behavior due to an increased concern for the immediate results of actions, enhanced by adverse living conditions of persistent poverty.

Our results indicate that SES significantly distinguishes the performance in future-oriented skills and language. While moderate-to-high effect sizes were found for all the cognitive variables studied, effect sizes were higher for EF. In fact, receptive language scores and EF (controlling for other future-oriented processes) are enough to determine SES group membership. These results are the first evidence that EF is related to SES to a greater extent than planning and delay of gratification, variables traditionally assessed in studies of poverty and child development (e.g., Raver et al., 2013). Reduced EF development may be a precursor of impaired executive functioning and self-control in children from low SES contexts since these processes demand, to some extent, envisioning and forecasting the future. Multiple linear regression models for future-oriented variables and the EF model yielded similar results after controlling by sex and receptive language, but maternal education was only significant in the EF model. This reveals a stronger association between SES and EF development when compared to the association between SES and the other future-oriented processes in this sample.

This research presents some limitations. First, since the selection of centers and families was made by convenience, recruiting a more diverse, representative sample would be desirable in future studies. Second, the sample size is small and consequently inadequate to perform further required analysis (e.g., moderation and mediation). Third, our design is cross-sectional; longitudinal studies are needed to determine if low SES is the cause of, for example, lower EF or CFC scores. Fourth, although PPVT is often used as a measure of general cognitive functioning, using other measures of cognitive competence (e.g., fluid intelligence measured by the Raven matrices) as control measures would contribute towards clarifying if the effects are specifically linked to language-related skills or to general intelligence. Finally, the Extadi-Gangoiti Scale was designed as an instrument that offers greater discriminative power for middle and upper-middle SES households in northern Spain (Arranz et al., 2014). Thus, our results may be affected by discriminative problems in population of low SES from Uruguay. Despite these limitations, these results may be useful to guide actions in psychological and educational practice, for example, by generating intervention programs to foster the development of EF in preschool education.

## Conclusions

This study investigated SES, parental consideration of future consequences, and quality of family environment’s association to EF development in early childhood and compared their effect regarding other future-oriented processes typically studied in the literature on poverty and child development. Our results suggest that SES significantly distinguishes children’s future-oriented skills and receptive language. Our data also suggests that the association of SES with EF is stronger in comparison to the association of SES with planning and delay of gratification. This study has implications for the understanding of how present-oriented behaviors in low SES contexts (Pepper & Nettle, 2017) may origin early in childhood as an adaptive response to structural and interactional factors associated to socioeconomic deprivation, and raises the need for further research on such associations and the kinds of interventions that should be encouraged.

## Data Availability

The datasets used and/or analyzed during the current study are available from the corresponding author on reasonable request.

## Abbreviations

CFC-I: Consideration of future consequences-immediate subscale
CFC: Consideration of future consequences
CFC-F: Consideration of future consequences-future subscale
DR: Delayed reward
EF: Episodic foresight
PPVT: Peabody Picture Vocabulary Test
SES: Socioeconomic status
ToH: Tower of Hanoi
